# Nuclear envelope remodelling during mitosis

**DOI:** 10.1016/j.ceb.2020.12.004

**Published:** 2021-06

**Authors:** Gautam Dey, Buzz Baum

**Affiliations:** 1 Cell Biology and Biophysics, European Molecular Biology Laboratory, 69117, Heidelberg, Germany; 2 Lab of Molecular Biology, Cambridge, CB2 0QH, United Kingdom; 3 Lab for Molecular Cell Biology, UCL, London, WC1E 6BT, United Kingdom

**Keywords:** Mitosis, Nuclear envelope, Nuclear division, Nuclear pore complex, Lamina, Eukaryogenesis

## Abstract

The defining feature of the eukaryotic cell, the nucleus, is bounded by a double envelope. This envelope and the nuclear pores within it play a critical role in separating the genome from the cytoplasm. It also presents cells with a challenge. How are cells to remodel the nuclear compartment boundary during mitosis without compromising nuclear function? In the two billion years since the emergence of the first cells with a nucleus, eukaryotes have evolved a range of strategies to do this. At one extreme, the nucleus is disassembled upon entry into mitosis and then reassembled anew in the two daughter cells. At the other, cells maintain an intact nuclear compartment boundary throughout the division process. In this review, we discuss common features of the division process that underpin remodelling mechanisms, the topological challenges involved and speculate on the selective pressures that may drive the evolution of distinct modes of division.

All eukaryotes organise their genome within a nucleus, a secure data storage centre and transcription hub, that is separated from the cytoplasm by a nuclear envelope [1]. The outer membrane of this nuclear envelope (NE) is continuous with the endoplasmic reticulum (ER) and is connected to the inner membrane of the NE by a series of pores formed at sites of high membrane curvature. These nuclear pores are scaffolded by nuclear pore complexes (NPCs), massive structures (in humans, around 110 MDa comprising over a thousand subunits) that regulate the shuttling of soluble material between nuclear and cytoplasmic compartments and membrane traffic between the inner and outer membranes [2]. As a result, although physically continuous with the outer NE and the ER, the inner NE forms a distinct, metabolically active [3] compartment with a unique lipidome [4] and proteome [5,6], whose identity is determined at least in part by its close physical proximity to the DNA [7].

Competing theories have been proposed to explain how the NE evolved during eukaryogenesis from the symbiosis of an archaeal and a bacterial cell, both of which were devoid of internal membranes. Such topological models aim to explain the unique organisation and asymmetry of the NE [8]. Collectively, they can be classified into ‘outside-in’ models, which envision the nucleus arising as a specialised extension of internal ER membranes that were brought together at pores to generate a continuous NE, and ‘inside-out’ models, which view the NE as a remnant of the original folded archaeal plasma membrane [9]. Whatever the path of eukaryogenesis, 2 billion years later, modern eukaryotic organisms cannot survive without a nucleus [10].

Nevertheless, the presence of a nucleus presents cells with a challenge. Each time a cell divides, it must duplicate and split its nuclear compartment into two. Although the mechanism by which this is done remains one of the least well-understood aspects of division, decades of experiments in animal and fungal models [11] have suggested that there are two, fundamentally different, ways to remodel the NE during mitosis:

1. Dismantle the nucleus and then reconstruct new compartments around the DNA when cells leave mitosis
2. Divide the entire intact nuclear compartment into two.

These strategies have long been termed ‘open’ and ‘closed’ mitosis, respectively. In recent years, however, the value of this binary classification has been brought into question as it has become increasingly clear that the majority of eukaryotes engage in a process that lies somewhere between these two theoretical extremes [12, 13, 14]. In fact, truly ‘open’ and ‘closed’ topologies may not even exist (Figure 1). Thus, remnants of the NE appear to interact with the mitotic spindle in the most open mitoses [15], while canonical examples of closed mitosis appear to exhibit transient, local, ‘opening’ during the final act of nuclear division [16,17] (Figure 1). In addition, closely related species [18,19] and even different cell types within the same organism [20] can exhibit remarkable differences in the degree of nuclear ‘opening’ during mitosis, and perturbing single genes can have a major impact on mitotic strategy [19,21] (Figure 2). Taken together, these various data highlight the plasticity of NE remodelling, mirrored by the plasticity of the NE itself [22]. This makes it possible to leave behind the old questions of classification and focus instead on the fundamental structural challenges that cells must overcome to successfully duplicate their nuclear compartment during mitosis. In doing so, one would aim to identify universal features of the process that are conserved across eukaryotes and unique adaptations restricted to certain lineages.

**Figure 1:**
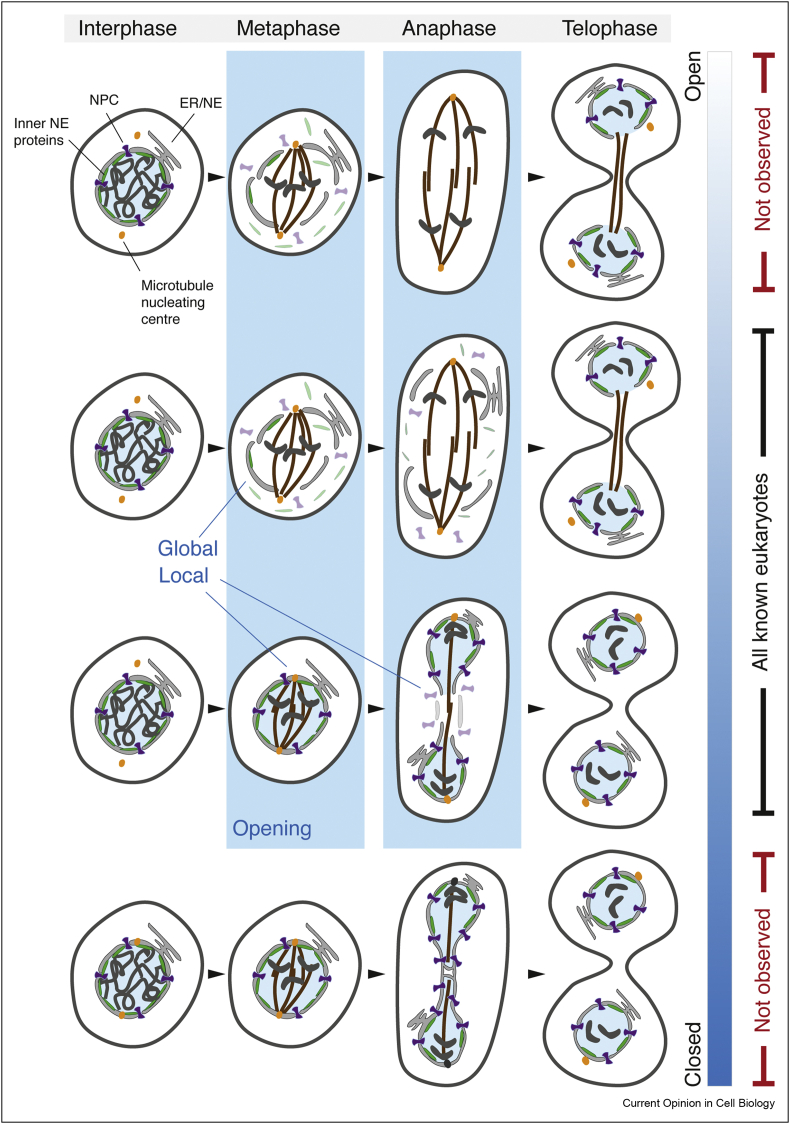
A schematic illustrating the full range of possible mitotic nuclear remodelling strategies, starting from completely open at the top to completely closed at the bottom. The compartment barrier is highlighted by labelling the nucleoplasm in blue and the cytoplasm in white; chromosomes are in dark grey and spindle microtubules in brown; other key cellular structures are labelled on the diagram. The two theoretical extremes have not been experimentally observed in any eukaryote to date. The blue bars indicate either local or global fenestration or ‘opening’ of the nuclear envelope, leading to a disruption of the barrier between the nucleoplasm and cytoplasm. In closed or semiclosed mitosis, the first local opening event allows the microtubule-nucleating centres to access the nucleoplasm; the second local opening drives nuclear division.

**Figure 2:**
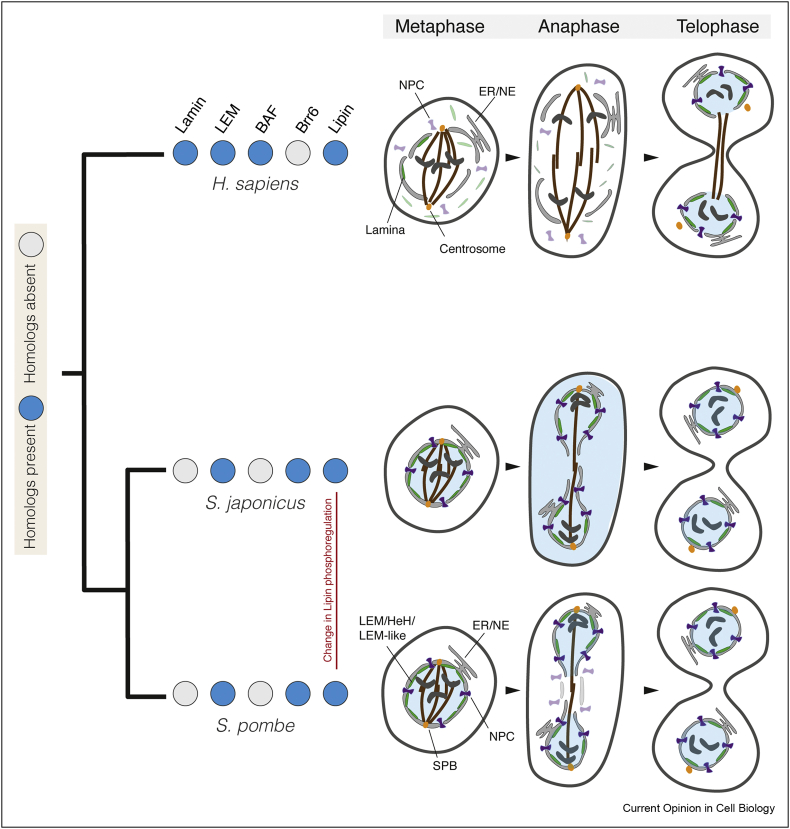
Plasticity in nuclear remodelling across well-studied model systems. The compartment barrier is highlighted by labelling the nucleoplasm in blue and the cytoplasm in white; chromosomes are in dark grey and spindle microtubules in brown; other key cellular structures are labelled on the diagram. The presence (blue) or absence (grey) of homologues of an illustrative subset of the key protein or domain families involved in controlling nuclear remodelling is shown as a phylogenetic profile (filled circles) for each species. Differences in mitotic mode (closed or open) can be observed at large evolutionary distances, for example, between humans and fission yeast (illustrated by distance on the tree and low phylogenetic profile similarity), but also between closely related species, for example, *S. pombe* and *S. japonicus* (illustrated by proximity on the tree and high phylogenetic profile similarity). A divergence in lipin phosphorylation during mitosis [19] is thought to be responsible for the tearing of the nuclear envelope and loss of integrity in *S. japonicus* mitosis that is not observed during local NE breakdown [16,17] in its sister species *S. pombe*.

## Prophase: compartment mixing

Eukaryotes generally rely on a microtubule-based spindle to segregate their chromosomes and cytoplasmic organelles [23] and, in many cases, to position the cleavage furrow [24]. Because microtubule-nucleating centres and the pool of tubulin itself play important roles in organising the interphase cytoplasm, the relevant proteins are excluded from the nucleus for most of the cell cycle [25]. This changes upon entry into mitosis, when tubulin and the microtubule-nucleating machinery need to gain access to the nucleus to construct a spindle. In all eukaryotes, this requires a loosening of the compartment barrier to enable a degree of mixing between the cytoplasm and nucleoplasm. Even in an open mitosis, this appears to be a tightly regulated process. Thus, tubulin is imported into the nucleus at the onset of NE disassembly [26,27], a process that is triggered by changes in NPC composition, structure and transport properties at the onset of prophase, downstream of the mitotic kinases CDK1 and PLK1 [28]. Importantly, this is a common feature of both open and closed mitoses [29].

If the microtubule-nucleating centre is not already embedded in the NE, as it is in budding yeast [30], the poles of the spindle or spindle microtubules must penetrate the NE through local or global envelope remodelling for them to gain access to the kinetochores of the condensed mitotic chromosomes. In an animal cell open mitosis, this is made possible through the phosphorylation and stepwise disassembly of NPCs and the NE [31] (Figure 2). In budding and fission yeasts, although this happens at different points in the cell cycle, the spindle pole body is imported through a transient pore in the NE in a poorly understood process [30] regulated by nucleoporins, SUN/KASH proteins [32] and the protein Brr6 [33] and likely requires subsequent envelope sealing (Figure 2).

## Metaphase: decoupling the DNA and spindle from the membrane

In metaphase, the DNA must become completely uncoupled from the overlying NE to freely segregate and partition into the two daughter nuclei at the end of mitosis [34]. This is the case in all eukaryotes. If this does not happen, such as when the function of NE-localised REEP proteins is compromised [35], serious chromosome segregation, cell division and nuclear morphology defects ensue [34]. The failure of DNA to disengage from nuclear pore complexes can also lead to its mis-segregation [36].

In a closed or partially closed mitosis, the architecture of the NE must somehow persist in the absence of attachments to DNA. Conversely, during an open or semiopen mitosis, the loss of contacts between the DNA and the overlying inner NE, together with loss of laminar integrity [37], likely contributes to the breakdown in coherence of the double membrane compartment barrier. It is important that the ensuing compartment mixing does not permit cytoplasmic structures, including membranes and organelles, to get in the way of spindle assembly or chromosome separation. Membranes derived from the NE and ER [31], as well as a poorly understood ‘proteinaceous matrix’ [38], function to ensure that cells retain a privileged nuclear space despite having lost their continuous NE. This has been observed in dividing HeLa cells (Figure 2) — a classic model of open mitosis — and in a variety of other systems without a fully intact nuclear compartment [39].

## Anaphase: partitioning

In both open and closed mitosis, once the chromosomes are properly attached to spindle microtubules and the spindle assembly checkpoint is satisfied, the clipping of the cohesive bridges that connect sister chromatids initiates anaphase [40]. Sister chromatids are then free to move to opposite poles of the spindle through the action of microtubule disassembly and the movement of kinetochore-anchored dynein motors towards the spindle poles, as spindle poles separate [23].

Importantly, although this process separates the DNA, cells must also find a way of coupling chromosome segregation to duplication of the nuclear compartment. Thus, as cells exit mitosis, the double membrane of the NE must be split into two without compromising the integrity of the ER lumen, which is topologically equivalent to the cell exterior. This requires NE expansion driven by ER lipid synthesis [41] — without which the NE can rupture — as seen in the fission yeast *S. japonicus*, where it leads to the transient loss of the compartment barrier at the site of division [18,19] (Figure 2). In most cells, however, division of the nuclear compartment is triggered by the disassembly of nuclear pores — sites at which the inner and outer NE wrap around to meet each other. This leads to loss of the nuclear compartment boundary and to fenestration of the envelope. In animal cells, NPC disassembly is initiated at the onset of mitosis [42,43], as it is in the unusual partially open mitosis of *Ustilago maydis* [44,45]. In the closed mitosis of the fission yeast *Schizosaccharomyces pombe*, where spindle elongation drives the formation of intact daughter nuclei connected only by a narrow bridge, nuclear division requires remodelling of the NE at a single site — the bridge midzone [16]. At the centre of the bridge, a subset of NPCs disassemble in a process analogous to that occurring at prophase in animal cells, fenestrating the membrane and exposing the spindle to the cytoplasm [16,17] as part of a local NE breakdown (Figure 2). As this makes clear, it is the timing and localisation of this NPC disassembly that determine whether a mitosis is more open or closed. However, it is not yet known how these differences are regulated.

## Telophase and cytokinesis: compartment demixing

Once the continuous NE has been compromised, the ER and/or NE must be segregated between daughter cells at cytokinesis [46,47] — a process that requires the ER to be reshaped [48]. The compartment barrier must then be re-established. In an open mitosis, where there is extensive mixing of the nucleoplasm and cytoplasm, this requires the active exclusion of the cytoplasm — achieved in part by the surfactant-like Ki-67 at the surface of chromosomes [49]. As nuclei reform, traffic across nuclear pores, driven by Ran-GTP, must then do the work of demixing the nucleoplasm and cytoplasm [50]. In *S. pombe* closed mitosis, the compartment barrier is maintained during local NE breakdown at least partially by the inner nuclear component Les1 [16], limiting the portion of NE that needs to be sealed to the tips of the stalk membranes and sites of spindle pole body insertions.

Across all systems studied thus far, this process of membrane sealing is a result of the action of the ESCRTIII system acting in coordination with the inner NE protein Lem2/LEM2 [∗51, ∗52, 53, 54, 55, ∗56]. In mammalian cells, ESCRTIII polymers and LEM2 act together with the microtubule disassembly factor Spastin to remodel and seal the envelope as spindle microtubules are disassembled [54,55]. In *Drosophila* cells, this entire process has been reported to proceed from the nuclear poles towards the midcell [57]. At the same time, as the nuclear compartment is re-established, functional NPCs must be assembled and inserted into the double envelope [58]. In many systems, this process is coordinated with the reassembly of the nuclear lamina [59,60], the disassembly of the spindle and the initiation of cytokinesis — a careful choreography that can be thrown into disarray if chromosomes have not segregated properly [61,62].

## Outlook

The discovery of common themes and molecular mechanisms (ESCRTIII, NPC disassembly) uniting open and closed nuclear remodelling strategies makes it easier to define the conserved aspects of the process of nuclear division. It also raises its own questions. If the regulatory differences between open and closed mitosis are relatively superficial, why do cells choose one remodelling strategy over the other? One major difficulty in addressing this question lies in interpreting the likely selective pressures arising from the conflicting roles of the nucleus during interphase and mitosis.

The interphase NE is essential for eukaryotic cell survival. It is the barrier that separates the cytoplasm and nucleoplasm and that enables RNA processing before translation. Thus, even a transient loss of the nuclear compartment boundary in interphase can have disastrous consequences for a eukaryotic cell, for example, by exposing the DNA to cytoplasmic nucleases [63].

At the same time, the nuclear compartment must be duplicated at division — a process that, as detailed earlier, relies on breaking the compartment barrier, at least transiently. Because of this, cells with an open mitosis must ensure that the DNA is adequately protected from cytoplasmic nucleases when the compartment boundary dissolves and must quickly re-establish the compartment boundary upon mitotic exit. Furthermore, while chromosome segregation and cell division may be relatively easy to coordinate in an open mitosis, as cells exit mitosis, the NE and NPCs must now be reassembled around the segregated DNA without losing stray chromosomes [59,64]. This would seem to be even more challenging in syncytia, where the ER must be partitioned between a large number of nuclei, something that may require additional mechanisms to limit contact between spindles [65].

Conversely, although freed from the constraints imposed by compartment mixing, cells undergoing a closed mitosis must ensure that the polarity and structure of the NE is maintained even in the absence of contacts between the chromatin and the NE and NPCs. Furthermore, the NE in cells undergoing a closed mitosis has to survive contortions in shape, area and shear as nuclei are remodelled to give rise to two daughter nuclei [11]. This in turn may constrain the size of the spindle. If so, one would expect an increase in genome size to be associated with a move to a more open mitosis. In addition, cells undergoing a closed mitosis have to coordinate nuclear division with organelle segregation and cytokinesis, a feat that might involve complex cross-compartment choreography — as is the case in budding yeast cells that require additional machinery to ensure that the anaphase spindle bridges the bud neck beforecell division [66]. Finally, even in cases of closed mitosis, the nucleus must be transiently and locally permeabilised either once or twice — first to insert the microtubule organising centre into the envelope, if it is not already embedded in the NE, and then again to actually split the nucleus — all the while keeping the nuclear compartment intact and insulated from the local permeabilisation process. Thus, the benefits of a closed mitosis may not be as great as were once imagined.

Speculatively, then, a need for greater nuclear autonomy and protection from cytoplasmic threats could shift the balance in favour of a closed mitosis. In contrast, an open mitosis would offer an easier way to coordinate chromosome segregation with the segregation of cytoplasmic organelles and cytokinesis, while also providing additional degrees of freedom with respect to spindle geometry and genome size.

Understanding how such selection pressures have acted to shape the mitotic strategies of living species will depend on identifying organisms in which the modes of nuclear division are flexible and depend on cell state (e.g., developmental stage or environmental conditions) and/or on reconstructing the evolutionary trajectories that led up to specific modes of nuclear division. In the latter case, this will require a more comprehensive description of nuclear remodelling mechanisms across poorly studied eukaryotes. Based upon the current data, although a change in the mode of nuclear division appears straightforward to trigger or evolve [12], in all known examples, this appears to occur in one direction (closed to open). In these cases, it will be interested to determine how cells then compensate for the additional compartment mixing — something that can be directly tested. While this might suggest an early origin for closed mitosis, the case is far from clear [9].

While such questions can be hard to answer, gaining a clearer picture of the likely evolutionary history of the nuclear compartment and nuclear division should be seen as much more than an exercise in the study of the history of life on earth. A better understanding of the evolution of nuclear division would be expected to have far-reaching implications for biology today: for example, by shedding light on the prevalence of syncytial divisions, by identifying ways of selectively killing parasites such as *Plasmodium* and *Giardia* which rely on closed nuclear division [67,68] and understanding the sensitivity of cells to transient nuclear rupture or delayed nuclear reformation at division. Fortunately, given recent advances in microscopy (including both live cell imaging and electron tomography) and in sampling the diversity of eukaryotic life, we might not be that far from a deeper understanding of the origins and core functional roles of the one organelle that all eukaryotes share.

## Author contributions

Both authors contributed equally to the conceptualisation, content and drafting of this review.

## Conflict of interest statement

Nothing declared.
